# Ursids evolved dietary diversity without major alterations in metabolic rates

**DOI:** 10.1038/s41598-024-55549-w

**Published:** 2024-02-27

**Authors:** A. M. Carnahan, A. M. Pagano, A. L. Christian, K. D. Rode, Charles T. Robbins

**Affiliations:** 1 School of Biological Sciences, Washington State University, Pullman, WA 99164-4236 USA; 2 U. S. Geological Survey, Alaska Science Center, Anchorage, AK 99508 USA; 3 Department of Rangeland, Wildlife and Fisheries Management, Texas A&M University, College Station, TX 77843 USA; 4 School of the Environment, Washington State University, Pullman, WA 99164-2812 USA

**Keywords:** Ecology, Evolution, Physiology, Evolutionary ecology

## Abstract

The diets of the eight species of ursids range from carnivory (e.g., polar bears, *Ursus maritimus*) to insectivory (e.g., sloth bears, *Melursus ursinus*), omnivory (e.g., brown bears, *U. arctos*), and herbivory (e.g., giant pandas, *Ailuropoda melanoleuca*). Dietary energy availability ranges from the high-fat, highly digestible, calorically dense diet of polar bears (~ 6.4 kcal digestible energy/g fresh weight) to the high-fiber, poorly digestible, calorically restricted diet (~ 0.7) of giant pandas. Thus, ursids provide the opportunity to examine the extent to which dietary energy drives evolution of energy metabolism in a closely related group of animals. We measured the daily energy expenditure (DEE) of captive brown bears in a relatively large, zoo-type enclosure and compared those values to previously published results on captive brown bears, captive and free-ranging polar bears, and captive and free-ranging giant pandas. We found that all three species have similar mass-specific DEE when travel distances and energy intake are normalized even though their diets differ dramatically and phylogenetic lineages are separated by millions of years. For giant pandas, the ability to engage in low-cost stationary foraging relative to more wide-ranging bears likely provided the necessary energy savings to become bamboo specialists without greatly altering their metabolic rate.

## Introduction

Quantifying and understanding the drivers of energy expenditure have been a major focus of physiologists and ecologists for decades^1,2^. Energy expenditures can be quite variable because at any one time they are determined by both proximal conditions (e.g., thermal environment, level of activity, growth rate, or amount of food consumed) and long-term evolutionary forces (e.g., life history characteristics including food habits)^3^. Recently, two groups measuring daily energy expenditure (DEE) of giant pandas (*Ailuropoda melanoleuca*) produced conflicting results even though both concluded that energy conservation is an important life strategy. One group reported that giant pandas have DEE (41 ± 12 kcal/kg^0.75^/day) similar to the three-toed sloth (*Bradypus variegatus*) and, therefore, much lower than virtually all other terrestrial mammals, including koalas (*Phascolarctos cinereus*) and echidnas (*Tachyglossus aculeatus*)^4^. Those results were contradicted by others who reported DEE three times higher (122 ± 51 kcal/kg^0.75^/day)^5,6^. Although housing and diets varied from captive to free-ranging and from only bamboo to mixtures of bamboo and human-type foods, neither group suggested or reported major differences in DEE due to diet or housing^4–6^. However, the latter group^5,6^ suggested that differences in activity might explain the differences in DEE within giant pandas and between giant pandas and other ursids, but that idea was not tested beyond noting that polar bears would have greater energy expenditures for travel and, potentially, thermoregulation than giant pandas^6^.

The family Ursidae consists of eight species that were found historically on all continents except Australia and Antarctica. Of the extant bears, the lineage that led to giant pandas diverged the earliest at 12 to 19.5 Mya whereas the most recent divergence was that of polar bears (*Ursus maritimus*) and brown bears (*U. arctos*) at 3.4 Mya^7–9^. Diets of the eight extant species range from carnivory (e.g., polar bears) to insectivory (e.g., sloth bears, *Melursus ursinus*), omnivory (e.g., brown bears), and herbivory (e.g., giant pandas). Dietary energy availability ranges from the high-fat, highly digestible, calorically dense diet of polar bears (~ 6.4 kcal digestible energy/g fresh weight) to the high-fiber, poorly digestible, calorically restricted diet (~ 0.7 kcal digestible energy/g fresh weight) of giant pandas^10–14^. Because food habits and the degree of energy restriction have been suggested as possible determinants of variation in basal metabolic rates (BMR) across species, BMR and DEE of ursids could vary in a similar manner with polar bears having the highest and giant pandas or sloth bears the lowest^3–6,15–18^.

However, we hypothesized that all ursids for which data are currently available have similar mass-specific DEE when measured under similar conditions. We further hypothesized that productivity (e.g., rate of gain or amount of nutrients consumed) and distance travelled (km) should be major drivers of DEE because both can be relatively high-cost functions. These hypotheses are based on similarities in the costs of lying, standing, and traveling for both polar bears and brown bears when measured on a treadmill^18,19^, similarities in the characteristics of movement (i.e., form and velocity) and digestive anatomy and physiology amongst all ursids (i.e., carnivore-type digestive system, rapid rate of digesta passage, and lack of significant fermentation) relative to that of tree sloths^12,20–22^, and the tendency for all ursids to select low-protein macronutrient dietary ratios that should reduce variation in the cost of nutrient metabolism^23^.

To test these hypotheses, we used doubly labeled water (DLW) to measure DEE of captive brown bears housed in a relatively large, zoo-type enclosure. Also, we compiled and compared those values to the available DEE and energy intake data on ursids beyond the three giant panda studies^4–6^, including one additional study on giant pandas^13^, one study each on captive and free-ranging polar bears^10,11^, and two previous studies on captive brown bears^24,25^. The study on free-ranging polar bears^10^ measured both DEE and travel distances for each bear such that we could extrapolate their DEE to what it would be at the daily travel distances of giant pandas.

In a previous brown bear foraging study using the same facility, bears were able to meet most to all of their maintenance energy requirements through foraging on the herbaceous vegetation growing in the enclosure^26^. To further simulate the intakes of wild bears through the seasons in this study, we provided various amounts of additional food to create differing levels of satiation, gain, heat increment of nutrient metabolism, and potentially activity and travel distances that would affect DEE. Thus, we were able to compare the energetics of bears with feeding habits that range from herbivory to omnivory and carnivory and that have daily travel distances ranging from ≤ 0.6 to 37.8 km/day^4–6,10^.

## Results

**Figure 1:**
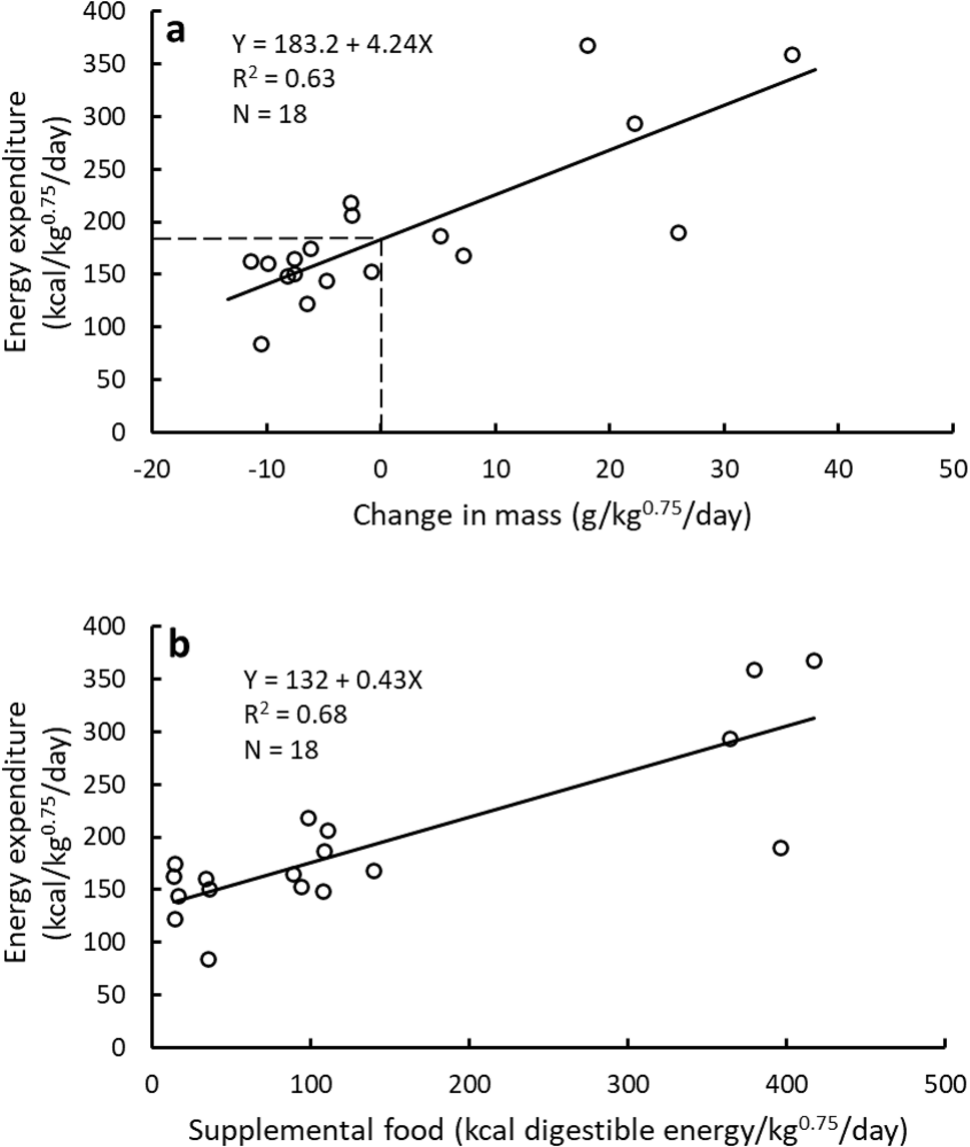
Relationships between change in mass and daily energy expenditure (**a**) and amount of supplemental food consumed and daily energy expenditure (**b**) in seven captive brown bears that had 24-h/day access to a 0.56 ha enclosure where they could forage on abundant, immature white clover and grasses. Measurements occurred on all seven bears in the spring and fall, but only on four of the seven bears during the summer (Table 1).

**Figure 2:**
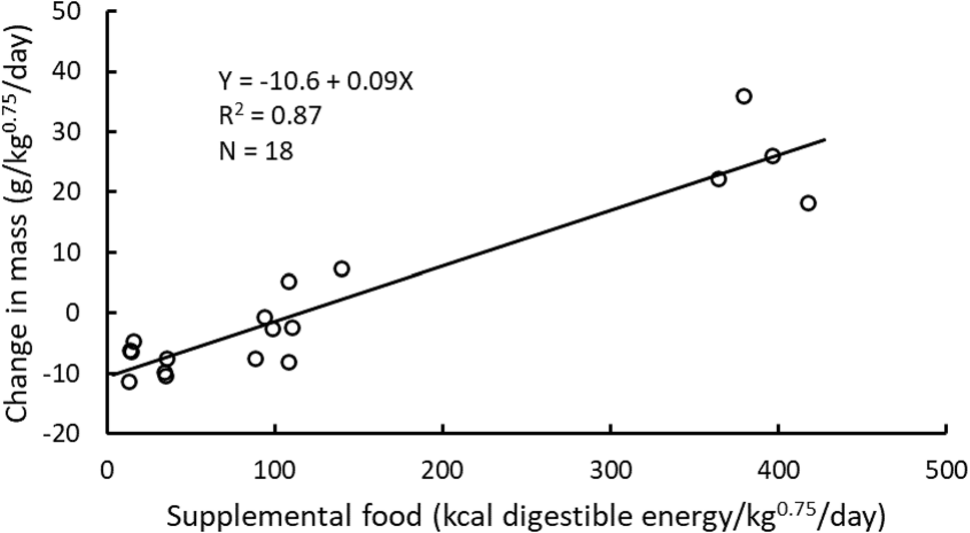
The relationships between the amount of supplemental food consumed and the change in mass by captive brown bears used to measure daily energy expenditure.

Daily energy expenditures (DEE) of captive brown bears housed in a relatively large enclosure averaged 191 ± 75 kcal/kg^0.75^/day (range 84–368) and were correlated with rates of mass change (F_(1,16)_ = 30.66, *p* < 0.001; Table 1, Fig. 1a) and the amount of supplemental food consumed (F_(1,16)_ = 34.57, *p* < 0.001; Fig. 1b). Rates of mass change averaged 2 ± 14 g/kg^0.75^/day (range − 11 to 36 g/kg^0.75^/day) and increased with the amount of supplemental food consumed (Fig. 2). Based on the relationship between DEE and rate of mass change, the maintenance cost at zero gain was 183 kcal/kg^0.75^/day (Fig. 1a). Grazing on the herbaceous vegetation growing in the enclosure accounted for 72% of that maintenance energy cost [i.e., Y-intercept of Fig. 1b at zero supplemental food (132 kcal/kg^0.75^/day) divided by the maintenance cost (183 kcal/kg^0.75^/day)]. Daily activity averaged 48 ± 10% of the day (range 30–61%, Table 1), and there was no significant relationship between DEE and activity (F_(1,16)_ = 1.558, *p* = 0.230).

**Table 1:** Characteristics of the brown bears used in each study and study results.

Bear	Sex	Mass (kg)	Age (years)	Season	Daily energy expenditure (kcal/kg^0.75^/day)	Rate of gain (g/kg^0.75^/day)	Supplemental food (kcal DE/kg^0.75^/day)	% Active	Length of study (days)
John	Male	246	16	Spring	144	-4.7	16	31	14
Frank	Male	246	16	Spring	186	5.2	109	35	14
Luna	Female	149	15	Spring	122	-6.5	15	49	14
Kio	Female	132	13	Spring	174	-6.2	14	54	14
Peeka	Female	132	13	Spring	153	-0.8	94	55	14
Willow	Female	105	3	Spring	163	-11.4	13	61	14
Zuri	Female	101	3	Spring	165	-7.6	89	59	14
John	Male	230	16	Summer	84	-10.5	35	30	11
Frank	Male	225	16	Summer	168	7.2	140	40	11
Peeka	Female	135	13	Summer	150	-7.6	36	50	12
Zuri	Female	108	3	Summer	160	-9.9	34	57	13
John	Male	242	16	Fall	190	26.0	396	36	7
Frank	Male	231	16	Fall	148	-8.2	108	47	7
Luna	Female	183	15	Fall	293	22.2	364	48	8
Kio	Female	156	13	Fall	359	36.0	379	47	8
Peeka	Female	151	13	Fall	218	-2.6	98	54	8
Willow	Female	138	3	Fall	368	18.1	417	60	8
Zuri	Female	121	3	Fall	206	-2.5	110	54	10

## Discussion

Significant correlations between DEE and rate of mass change or supplemental food consumed reflect the role of nutrient metabolism as a driver of energy expenditure, particularly in this case when supplemental intake ranged from 13 to 417 kcal digestible energy (DE)/kg^0.75^/day (Table 1). The lack of a correlation between activity and DEE likely occurred because activities can range from low-cost postural adjustments to high-cost running and, therefore, a general activity measure may not be closely tied to energy expenditure. Thus, even though the activity levels in captive brown bears (range 30–61%, mean 48 ± 10%), free-ranging giant pandas (~ 49 ± 5%), captive giant pandas (~ 33 ± 7%), and free-ranging polar bears (13–60%, 34 ± 7%) cover a similar range, their travel distances and DEE can be very different^4,10^.

**Figure 3:**
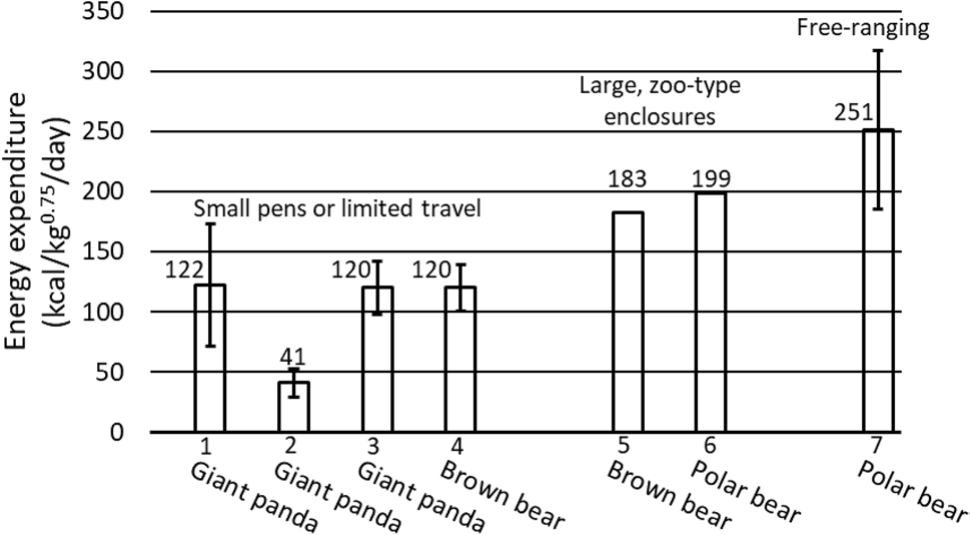
Relationships in ursids between daily energy expenditure (DEE), metabolizable energy intake, and travel distances. The values from left to right include (1) DEE of captive giant pandas housed in large enclosures, feeding on bamboo, and measured with DLW^5,6^, (2) DEE of captive and wild giant pandas feeding on bamboo when measured with DLW^4^, (3) metabolizable energy intake of captive giant pandas during bamboo digestion studies that were likely at or near maintenance^13^, (4) metabolizable energy intake by captive brown bears at maintenance and housed in small 3 X 8-m pens where travel was restricted^11,24,25^, (5) metabolizable energy intake by captive brown bears at maintenance that were housed in large zoo-type enclosures (current study), (6) metabolizable energy intake by captive polar bears at maintenance that were housed in large zoo-type enclosures^11^, and (7) DEE of free-ranging polar bears hunting seals on Arctic ice in the spring that was measured with DLW^10,37^.

DEE at maintenance (i.e., zero mass change) in captive brown bears determined with DLW (183 kcal/kg^0.75^/day, current study) is similar to the metabolizable energy intake at maintenance occurring in captive polar bears (199 kcal/kg^0.75^/day)^11^ when both had access to relatively large, zoo-type enclosures (brown bears, 0.56 ha; polar bears, 0.62 ha) and optimum dietary macronutrient ratios (Fig. 3). However, these levels of energy expenditure are ~ 60% higher than that of brown bears (120 ± 19 kcal/kg^0.75^/day) fed at maintenance with similar macronutrient ratios but confined to small pens (3 × 8-m) where travel was restricted^24,25^. This latter level of DEE in captive brown bears does not differ (t = − 0.075, df = 11, *p* = 0.941) from that measured for captive giant pandas during the summer and autumn (122 ± 51 kcal/kg^0.75^/day) by the group suggesting that giant pandas do not have exceptionally low metabolic rates^5,6^ (Fig. 3). Although the giant pandas were housed in large enclosures (0.77–49.4 ha)^6^ with abundant bamboo where natural foraging activities might have led to elevated DEE as occurs in captive brown bears, both captive and free-ranging giant pandas travelled ≤ 0.6 km/day because most of their active time was spent in low cost, stationary foraging^4–6,27^.

Further support for this level of DEE by giant pandas (122 ± 51 kcal/kg^0.75^/day)^5,6^ rather than the much lower estimate (41 ± 12 kcal/kg^0.75^/day)^4^ can be found in bamboo digestion studies in which ad libitum bamboo was provided with few additional energy supplements (i.e., bamboo providing 98.4 ± 0.7% of energy consumed)^13^. The metabolizable energy intake (i.e., digestible energy times 0.95)^28^ in those studies averaged 120 ± 22 kcal/kg^0.75^/day (Fig. 3). While we do not know if the giant pandas used in any of the various studies^4–6,13^ gained or lost mass, the giant pandas used in the digestion studies were able to feed selectively as occurred in the field studies^4–6^, and the exceedingly poor energy digestibility of bamboo (26 ± 14%)^13,14^ likely minimized gain.

Several other giant panda digestion studies have been performed using mixed, zoo-type diets (i.e., National Zoo, Washington D. C.; Memphis Zoo, USA; and Anji County Exposition Park, China) with bamboo providing 79 ± 11% of the total dietary energy that was supplemented with more energy dense foods (e.g., corn bread, gruels of cooked grains, cat or dog food, fruits, and other supplements)^12,13,29^. Metabolizable energy intake in those studies averaged 221 ± 81 kcal/kg^0.75^/day (n = 10), which should have resulted in either or both increased activity or a modest gain based on a maintenance level of ~ 120 kcal/kg^0.75^/day. However, if the maintenance energy expenditure in giant pandas is 41 ± 12 kcal/kg^0.75^/day^4^, a metabolizable energy intake of 221 ± 81 kcal/kg^0.75^/day (i.e., 5.4 times higher) should have resulted in the massive gains and obesity characteristic of brown bears consuming unlimited salmon or polar bears feeding on abundant seals, which seems unlikely. Thus, the metabolizable energy intakes during bamboo digestion studies (120 ± 22 kcal/kg^0.75^/day)^13^ and the higher estimates of DEE (122 ± 51 kcal/kg^0.75^/day)^5,6^ using doubly labeled water suggest that the giant panda DEE is similar to the mass-specific metabolizable energy intake at maintenance by brown bears (120 ± 19 kcal/kg^0.75^/day)^24,25^ housed in small pens and potentially other ursids when travel is restricted and significant gain is not occurring (Fig. 3).

**Figure 4:**
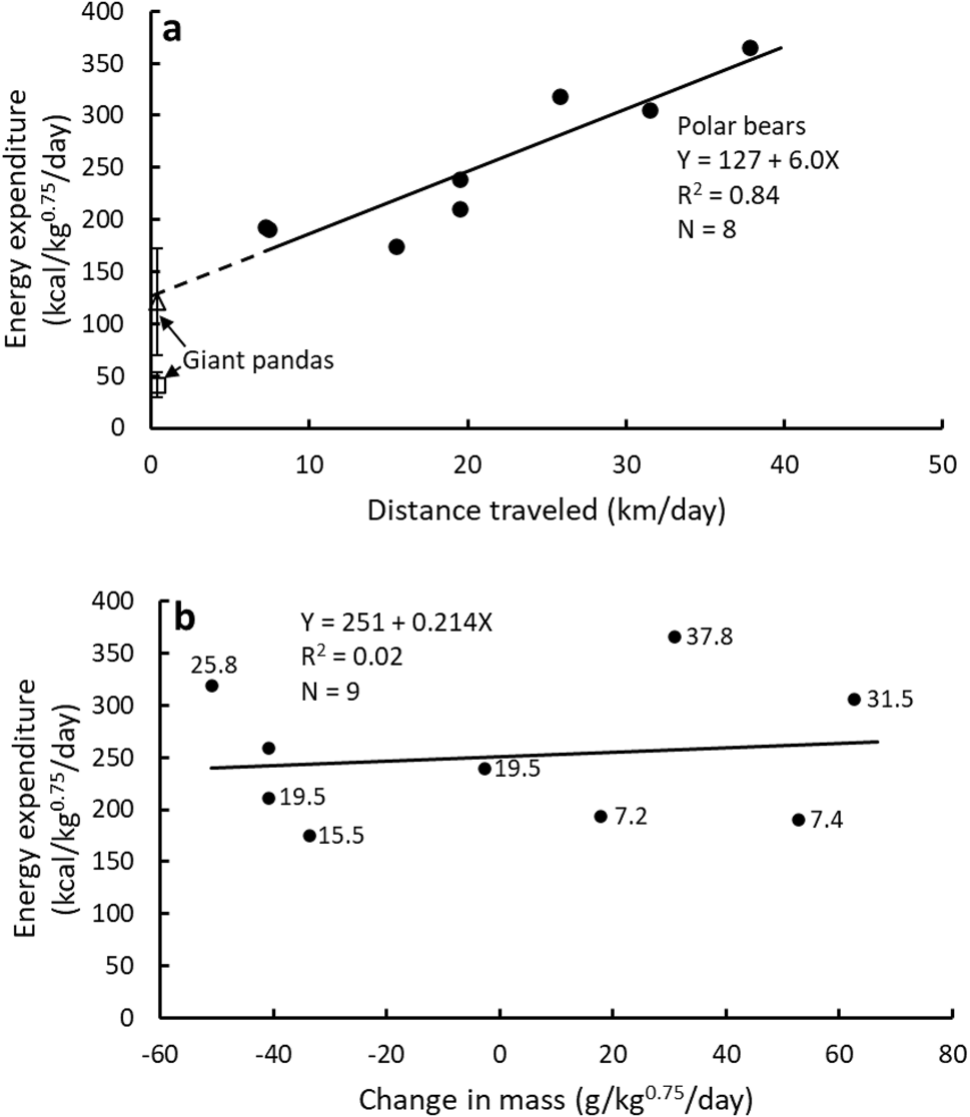
The relationships between daily energy expenditure (DEE) in free-ranging polar bears^10,37^ and either distance traveled or rate of mass change. The distance travelled regression is compared to the two estimates of DEE for giant pandas (open square 41 ± 12 kcal/kg^0.75^/day (n = 8)^4^, open triangle 122 ± 51 kcal/kg^0.75^/day (n = 8)^5,6^). The giant panda values are means ± 1 SD whereas the polar bear values are for individuals. The regression in (**a**) is for polar bears only. The value adjacent to each polar bear data point in (**b**) is the average travel distance (km/day) for that bear, except for one bear whose GPS collar failed.

The average DEE for free-ranging polar bears actively hunting seals on Arctic sea ice in the spring when measured with DLW (251 ± 66 kcal/kg^0.75^/day)^10^ ranges from ~ 32% higher than that of captive polar bears (199 kcal/kg^0.75^/day) and brown bears (183 kcal/kg^0.75^/day) at maintenance when housed in relatively large zoo-type enclosures to ~ 209% higher than that of brown bears at maintenance when housed in small pens (120 ± 19 kcal/kg^0.75^/day)^11,24,25^ (Fig. 3). These differences in DEE due to housing likely reflect the increasing cost of travel as the free-ranging polar bears traveled from 7.2 to 37.8 km/day, which accounted for 84% of the variation in their DEE (Fig. 4a)^10^. The cost of travel was such an important determinant of DEE that rate of gain as an index of nutrient intake had no effect on DEE (Fig. 4b), although one must always be concerned about the inability to control for variation in gut-fill when determining mass and therefore rate of change by wild bears in short-term studies. However, the maintenance cost at zero gain (Fig. 4b; 251 kcal/kg^0.75^/day) was the same as their average DEE (251 ± 66 kcal/kg^0.75^/day).

When the mass-specific DEE of free-ranging polar bears is extrapolated to the daily travel distances of giant pandas (≤ 0.6 km/day) (Fig. 4a), that estimated DEE (~ 127 to 130 kcal/kg^0.75^/day) is within the variation of the higher estimate for giant pandas (122 ± 51 kcal/kg^0.75^/day)^5,6^ determined with DLW, the metabolizable energy intake of giant pandas during bamboo digestion studies (120 ± 22 kcal/kg^0.75^/day)^13^, and the metabolizable energy intake at maintenance in brown bears housed in small pens (120 ± 19 kcal/kg^0.75^/day)^11,24,25^, all of which are well above 41 ± 12 kcal/kg^0.75^/day^4^. Thus, all active ursids likely have similar mass-specific DEE when travel distances and energy intake are similar even though their food habits differ dramatically and evolutionary divergences occurred millions of years apart. The one potential exception because of their unique life history is sloth bears, although previous estimates of their metabolic rates suggest that they may not be distinctly different from the other ursids^17^. Of course, when the above mass-specific DEE are expressed on a per animal basis and markedly different travel distances are included, DEE will be much higher in large, wide-ranging polar bears and brown bears than smaller, sedentary giant pandas.

While our results do not question the general concept that dietary specialization in an evolutionary context can lead to differences in metabolic rates^3,15,16^, our results suggest that the different diets of ursids may not be of over-riding importance in determining metabolism of active season ursids. For giant pandas the ability to engage in low-cost, stationary foraging alone may have provided the necessary energy savings for them to specialize on a low-energy density, bamboo diet without the need to depress BMR. However, any habitat alterations that increase travel distances and therefore DEE of giant pandas will likely decrease the available net energy and therefore their well-being. Finally, the recognition of the importance of travel and the cost of nutrient metabolism and mass gain^11,18,19,24,25^ in determining DEE of ursids could ultimately provide a basis to accurately estimate DEE of active ursids in many circumstances.

## Methods

### Animals

Brown bears were housed at the Washington State University Bear Research, Education and Conservation Center (Pullman, Washington, USA) in accordance with procedures approved by the Washington State Institutional Animal Care and Use Committee, protocol #04780. All trials were performed in accordance with the relevant guidelines of this committee. This study is reported in accordance with ARRIVE guidelines. The bears in the facility are active from mid-March to early November when hibernation begins. Bears of both sexes were used with ages ranging from 3 to 16 years (Table 1). Diet and feeding schedules were similar to those described previously^30^.

### Study design

In 2018 we conducted 18 trials with 7 bears lasting 8–14 days during three periods: May–June (late spring), July–August (summer), and September–October (fall) (Table 1). For the spring and fall we divided the bears into three groups composed of two adult males, three adult females, and two subadult females. The summer was restricted to two adult males, one adult female, and one subadult female. During each trial, bears were allowed 24-h access to a 0.56-ha yard where they could graze on early growth herbaceous vegetation. Bears also received supplemental feed (Mazuri Wild Carnivore Bear Maintenance Diet, Land O'Lakes, Inc., St. Louis, MO) at rates of digestible energy intake ranging from 33% in the spring to 620% in the fall of the metabolic rate determined by Y = 70M^0.75^, where M is mass in kg and Y is in kcal/day^31^. Digestible energy content of the commercial diet (4.18 kcal/g dry matter) was previously determined in 5-day total collection brown bear feeding studies. The additional food was provided to mirror intakes of wild bears occurring in different seasons and to create differing levels of satiation, growth, heat increment of nutrient metabolism, and potentially activity and travel distances that would affect DEE. Because we could not measure total food intake, we used growth rate as an index of total energy and nutrient intake.

All bears wore collars with tri-axial accelerometers (Actigraph™ GT3X + , Pensacola, FL, size = 4.6 cm × 3.3 cm × 1.5 cm, mass = 19 g) which recorded acceleration at ± 16 g and were housed in waterproof cast aluminum cases (Polycase, Avon, OH) attached to either the left or right side of the collar. Following each trial, the data were downloaded and extracted into raw.csv files using ActiLife 6 software (Actigraph, Pensacola, FL).

Bears also were monitored using video cameras mounted in each den, outdoor run, and outdoor exercise yard. Thus, the combination of video footage and Actigraph sensor data were used to train a random forest model to determine time spent in various daily activities (e.g., lying, standing, walking, foraging, and running) that could be related to energy expenditure^32^. Those times were further summarized into inactive (lying) and active (all other activities). We did not measure travel distances or rates of travel because of significant variation between individuals and activities (i.e., foraging to running), and darkness precluded 24-h observations.

### Doubly labeled water energy measurements

At the beginning of each doubly labeled water (DLW) trial, all bears were fasted overnight. An initial blood sample was drawn the next morning for background isotope measurements. Subsequently, bears were either orally dosed or injected with 1.5 g ^18^O (10%) and 0.1 g deuterium (^2^H, 99%) per kilogram body mass. Five bears that were trained for voluntary blood draws were orally dosed by adding diluted honey to the DLW. After the initial dose was consumed, the bottle containing the DLW was rinsed three times with dilute honey, and each rinse was fed to ensure the bear received the entire dose. Subsequent blood draws in these trained bears occurred at 4 h, 24 h, 7 days, and on the final day (i.e., 7–14 days).

Because two large males were not trained for voluntary blood draws and required very large volumes of DLW, they were anesthetized with a combination of Telazol® and dexmedetomidine^33^. Due to the volume and need to inject the dose intravenously, an appropriate amount of 20X tris buffered saline (TBS; Amresco LLC, Solon, Ohio, USA) was added to the DLW. This solution was purified by drawing it through a 0.2 µm filter into a syringe and weighed. After the background blood sample was drawn, the DLW was slowly injected into the cephalic vein. Following injection, blood was drawn into the syringe and flushed back into the cephalic vein three times to ensure the entire dose was given. An equilibrated blood sample was drawn 2 h later based on previous studies using both captive and wild brown bears, polar bears, and American black bears (*U. americanus*)^34,35^, and then the bears were reversed using atipamezole.

The length of the trials depended on the anticipated rate of water turnover and energy expenditure with the goal of ensuring that isotope levels remained above background at the end of each study. The shorter trials occurred during hotter weather when water turnover would increase and in the fall when higher food intakes occurred. Final blood samples were taken following an overnight fast. All blood samples were centrifuged, and serum samples were collected, frozen, and stored at – 80 °C. Serum samples were sent to Metabolic Solutions (Nashua, NH, USA) for DLW analyses. Daily energy expenditure (DEE) was calculated using the method of Speakman and Hambly^36^. At least 30 days passed between trials to ensure that isotope levels had returned to background levels.

### Comparisons to other ursid energy expenditures

Several other studies of ursid energy expenditure or energy intake have been published that can be compared to the current values^4–6,10–13,24,25,37^. The studies can be divided into two types: those using doubly labeled water (DLW) and therefore measuring CO_2_ production as an index of heat production and those doing feeding studies and measuring digestible energy intake. To compare energy values between studies, we needed to convert digestible energy intake into heat production that is equivalent to that measured using DLW. That occurs only at maintenance (i.e., zero mass change) and when digestible energy is corrected for urinary energy losses to determine metabolizable energy. Thus, we multiplied the digestible energy intakes at maintenance by the average metabolizable energy coefficient for ursids (i.e., 0.95) to correct for urinary energy losses^28^. Energy loss due to gas production from fermentation was assumed to be negligible because ursids are monogastrics, have no sites for significant fermentation, and have very fast rates of digesta passage^12,28^.

Because we were interested in comparing DEE and metabolizable energy intake across ursids of different adult size, we had to select an exponent of mass that would create overlap in the values between species. While we have previously used an exponent of 1 (i.e., per kg) to describe the cost of locomotion^18,19^ and because BMR across mammals is best described by mass to the ± 0.67 power^38,39^, scaling of DEE across mammals likely requires some intermediate exponent, such as 0.734 for all mammals or 0.77 for eutherians only^40,41^. Many of the ursid metabolic measurements that might help identify a correct exponent for such an interspecific comparison have been done during hibernation (e.g., brown bears and American black bears) or during long-term fasting or restricted feeding when polar bears are forced onto land during the ice-free season^42–46^. During hibernation in brown bears, even when temporarily fed, thousands of genes affecting metabolism are either up-regulated (e.g., lipolysis) or down-regulated (e.g., glucose uptake and glycolysis) in comparison to that occurring during the active season^30,47,48^, which suggests that metabolic rates determined during seasonal hibernation or fasting and restricted feeding are not representative of active season bear metabolism. Thus, because of the many unknowns and “considerable variation” in both BMR and DEE^38–41^, we decided to use the exponent 0.75 to compare DEE across ursids, which is intermediate to the exponents (0.734 and 0.77)^40,41^ previously used to describe DEE of mammals and is one that we’ve used in several previous studies^11,24–26^. However, should others want to express brown bear DEE in different formats, we provide the brown bear masses and respective energy expenditures for each study (Table 1).

### Statistical analyses

We used linear models using the statistical software R^49^ to explore relationships between DEE and growth rate, daily activity, and supplemental food consumed. To compare differences in DEE between ursids, we first tested for homoscedasticity using Fisher’s F-test because we had unequal sample sizes. We used Student’s two-sample t-test to determine if differences in mean DEE were significant when variances were homoscedastic and Welch’s t-test when variances were heteroscedastic. We accepted P ≤ 0.05 as a statistically significant difference. All means are reported ± 1 SD.

## Data Availability

The datasets generated during and/or analyzed during the current study are available from the corresponding author on reasonable request.
